# Electropolymerized Molecularly Imprinted Polypyrrole Film for Sensing of Clofibric Acid

**DOI:** 10.3390/s150304870

**Published:** 2015-02-26

**Authors:** Bianca Schweiger, Jungtae Kim, Young Jun Kim, Mathias Ulbricht

**Affiliations:** 1 KIST Europe Forschungsgesellschaft mbH, Campus E 7.1, 66123 Saarbrücken, Germany; E-Mails: schweiger@kist-europe.de (B.S.); tais@kist-europe.de (J.K.); 2 Lehrstuhl für Technische Chemie II, Universität Duisburg-Essen, Universitätsstraße 7, 45141 Essen, Germany; E-Mail: mathias.ulbricht@uni-due.de

**Keywords:** molecularly imprinted polymer, polypyrrole, clofibric acid, quartz crystal microbalance, electrochemical polymerization, amperometric sensor

## Abstract

Piezoelectric quartz crystals and analogous gold substrates were electrochemically coated with molecularly imprinted polypyrrole films for pulsed amperometric detection (PAD) of clofibric acid, a metabolite of clofibrate. Cyclic voltammetry data obtained during polymerization and deposited weight estimations revealed a decrease of the polymerization rate with increasing clofibric acid concentration. XPS measurements indicated that clofibric acid could be removed after imprinting with an aqueous ethanol solution, which was further optimized by using PAD. Zeta potential and contact angle measurements revealed differences between molecularly imprinted (MIP) and non-imprinted polymer (NIP) layers. Binding experiments with clofibric acid and other substances showed a pronounced selectivity of the MIP for clofibric acid *vs.* carbamazepine, but the response of MIP and NIP to 2,4-dichlorophenoxyacetic acid was higher than that for clofibric acid. A smooth surface, revealed by AFM measurements, with roughness of 6–8 nm for imprinted and non-imprinted layers, might be a reason for an excessively low density of specific binding sites for clofibric acid. Furthermore, the decreased polymerization rate in the presence of clofibric acid might not result in well-defined polymer structures, which could be the reason for the lower sensitivity.

## 1. Introduction

The preparation of cross-linked synthetic polymers with specific binding sites in the presence of template molecules is called molecular imprinting. Monomer and template molecules having complementary functional groups interact with each other through the formation of covalent or non-covalent bonds in the solvent, which acts as a porogen. After polymerization, the template is removed via washing steps leaving cavities, which fit to the geometrical and functional properties of the template. Template or similar molecules can bind again in these cavities. This approach was first used by Wulff to create “enzyme-analogue built polymers” [1]. In the non-covalent approach, interactions between functional groups of monomer and template molecules such as ionic interactions, hydrogen bonds, π–π interactions, and hydrophobic interactions are utilized [2].

MIPs can be used as antibody and receptor mimics, e.g., materials obtained by imprinting with theophylline and diazepame [3], for enzymatic catalysis, e.g., for mimicking the active center of the digestive enzyme chymotrypsin [4,5], and as biosensors. The recognition elements in biosensors usually consist of antibodies, enzymes or other biological receptors, which are immobilized on the sensor surface. When an analyte binds to the recognition element, the resulting physical change is transduced into a signal, which can be monitored. This can be a heat change (calorimetric), a change of optical properties (e.g., absorbance, fluorescence, chemiluminescence), a mass change (piezo-electric), or an electrochemical change. Electrochemical biosensors measure current (amperometric), voltage (potentiometric), conductance (conductometric), or impedance changes (impedimetric). For instance, enzymatic biosensors can be based on oxidoreductase enzymes coupled with amperometric detection, where a change of current as a result of electrochemical oxidation or reduction is detected, e.g., the electron transfer when glucose binds with glucose oxidase [6]. The replacement of natural receptors with MIPs could be advantageous for sensing purposes, because natural receptors are sensitive to environmental conditions (temperature, pressure, and pH). Also, MIPs can be used for substances, which have no natural receptor. Small organic molecules such as pharmaceuticals, pesticides, amino acids and sugars as well as larger organic compounds such as proteins, viruses, and even cells are examples for templates used for molecular imprinting [7].

**Figure 1:**
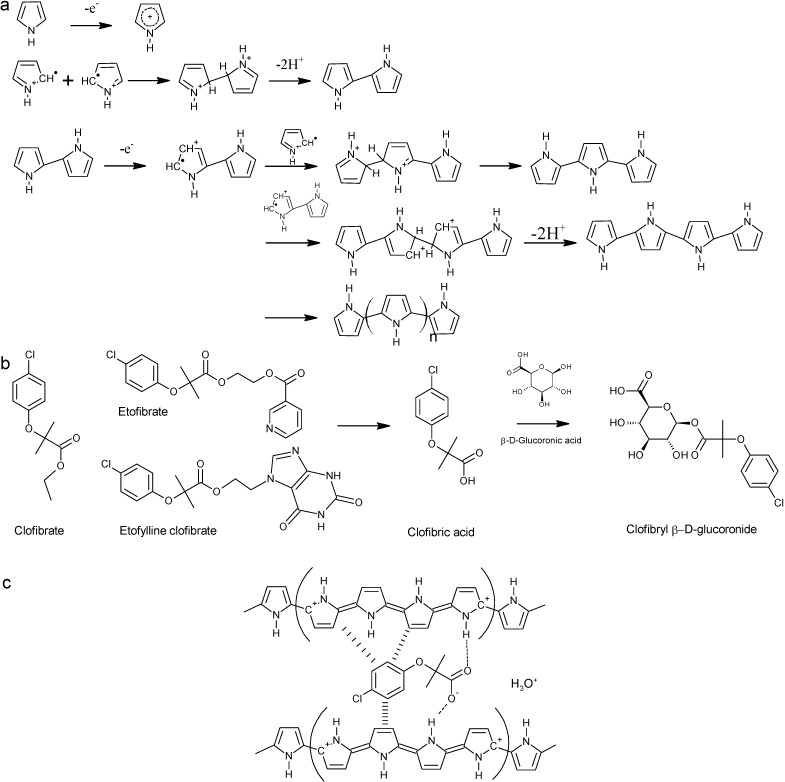
(**a**) Overview on polymerization reactions of pyrrole; (**b**) Precursors and metabolic product of clofibric acid; (**c**) Non-covalent interactions of clofibric acid with polypyrrole.

Conducting polymers show metal-like behavior due to conjugated double bonds, e.g., polyacetylene, polypyrrole, polythiophene, polyphenylenevinylene, or polyaniline. The polymerization can be done in organic solvents or aqueous solutions at room temperature, which is advantageous for the imprinting of biomolecules, because denaturation and conformational changes can be avoided [8]. Polypyrrole belongs to the first routinely electrochemically synthesized polymers [9]. It is polymerized by anodic oxidation on the working electrode. During the initial step, pyrrole monomers are oxidized to radical cations, which couple to dimer cations (Figure 1a). Proton elimination leads to neutral dimers. Early research proposed a propagation mechanism, where oxidized dimers couple with monomeric radical cations to build oligomers, which themselves couple with radical cations. Later research proposes that coupling of monomeric radical cations with each other is dominating due to high rate constants [10]. The oxidized dimers couple then again with each other leading to tetramers and then to octamers. Additional coupling reactions, which result in trimers or hexamers, may occur with increasing monomer concentration. Examples of templates used for polypyrrole imprinting include caffeine [11,12,13], glutamic acid [14], sodium taurocholate [15], L-aspartic acid [16], adenosine, inosine and ATP [17], adeoxynivalenol [18], dopamine [19,20], quercetin [21], clopidol [22], gliclazide [23], sulfanilamide [24], and sulfadimethoxine [25].

For preparation of conducting imprinted polymer films, electrochemistry techniques such as galvanostatic, potentiostatic and cyclic voltammetry-based deposition were used, which allow the control of the film thickness. In combination with piezoelectric quartz crystals as working electrodes the observation of the mass deposition is possible [11,26]. To detect the binding of template molecules, pulsed amperometric detection (PAD) was applied. In combination with conducting polymers, it can be used to detect electroinactive anionic molecules with flow injection analysis. When a positive potential is applied to the polymer, it is oxidized and negatively charged molecules from the solution penetrate into the polymer, which gives an anodic current peak in the flow system. A following lower potential reduces the polymer and the negative ions are expelled from the polymer, which results in cathodic current [27,28]. This method was used for neutral molecules by Ramanaviciene *et al.* in combination with conducting imprinted polymers for the detection of caffeine and bovine leukemia virus glycoproteine gp51 [29,30]. Pyrrole was polymerized on a Pt electrode in the presence of caffeine or gp51 in aqueous solution by applying 20 potential pulses between 950 and 350 mV. After template removal, the binding of the target molecule to the imprinted sites was detected by application of several potential steps and the sensor response was obtained by the peak difference of the current response. When neutral target molecules bind to the imprinted sites, the electron flow is reduced until saturation. PAD was also used for the detection of caffeine with an array of carbon nanotubes grafted with imprinted polypyrrole [12] and on Au electrodes [13]. The advantage of this method is the possibility to detect the binding of electroinactive substances to imprinted sites.

In this project, clofibric acid was used as a template. Clofibric acid is the pharmacologically active metabolite of the blood lipid regulators clofibrate, etofyllinclofibrate, and etofibrate. It was increasingly found during the last 30 years in waste waters, ground water, surface waters, and tap water [31], because it is hardly removed by waste water treatment plants. While about 2% of the original doses of clofibrate or clofibric acid were found to leave the body unchanged after 24 h [32], clofibric acid is mainly excreted as gluconoride from the body (Figure 1b). A cleavage of this molecule might occur in waste water treatment plants, which could add to the detectable amounts of clofibric acid in surface waters [31]. For the effective removal of chemical pollutants from waste water, adsorption processes are studied. With graphene oxide nanosheets, over 90% of clofibric acid were removed from acidic aqueous solutions [33]. Also, MIPs are explored for extraction and following analytical determination of pollutants, but also for their selective adsorption, and catalytic degradation [34]. Vinylpyridine-based imprinted polymers for the removal of clofibric acid from environmental water samples have been developed [35,36,37]. Also, a commercially available imprinted polymer for solid phase extraction of non-steroidal anti-inflammatory drugs was tested with clofibric acid [38].

Figure 1c shows possible non-covalent interactions between clofibric acid and polypyrrole which could be exploited during molecular imprinting. The aim had been to elucidate how efficiently the electropolymerization of polypyrrole could be used to obtain a MIP-based sensor for clofibric acid.

## 2. Experimental Section

### 2.1. Chemicals

Pyrrole was purchased from Sigma Aldrich and freshly distilled at 128 °C, flushed with nitrogen and stored in the dark at 4 °C to prevent oxidation. Clofibric acid, 2,4-dichlorophenoxy acetic acid (2,4-D), carbamazepine (CBZ), dimethylsulfoxide (DMSO), potassium nitrate (KNO_3_), potassium dihydrogen phosphate, sodium hydroxide, sodium p-toluenesulfonate, sulfuric acid (95%), hydrogen peroxide (30%), isopropanol, acetonitrile, methanol, and ethanol were used as received. Nitrogen gas (Alphagaz) was obtained from Air Liquide. Ultrapure water from a Millipore Milli-Q purification system was used for preparation of solutions. Solutions for polymerization and binding were filtered before use with 0.2 µm polypropylene syringe filters. Stock solutions of clofibric acid (1 mM) were prepared in KNO_3_ or phosphate buffer solution (pH 7.0) and kept refrigerated. Stock solutions of 2,4-D and CBZ (1 mM) were prepared in phosphate buffer solution (pH 7.0) and kept refrigerated. CBZ was first dissolved in 2 mL DMSO and filled up to 100 mL with buffer solution.

### 2.2. Instrumentation

Voltammetric measurements were performed with a potentiostat (Autolab PGSTAT 12, Metrohm/Eco Chemie, Utrecht, The Netherlands) with GPES software (Eco Chemie). Part of the coating experiments were done in a 10 mL beaker with gold coated glass wafers as working electrodes, a platinum wire as counter electrode and a Ag/AgNO_3_ electrode as reference electrode. QCM measurements were performed with a Q-Sense E1 system (Q-Sense, Biolin Scientific AB, Stockholm, Sweden) with an electrochemistry flow module (QEM 401), which was connected to the potentiostat. Sample solutions were introduced into the electrochemistry module with a peristaltic pump (IKA). Quartz crystal gold sensors (4.95 MHz, AT cut) were used as working electrode (exposed area 1.131 cm^2^), a platinum plate as counter electrode, and a Dri-REF Ag electrode (WPI, Sarasota, FL, USA) as reference electrode. Before every experiment, water, isopropanol, and water were pumped subsequently through the cell to remove contaminations and trapped air bubbles. Wafers and quartz crystals were cleaned with a 2:1 (v/v) mixture of sulfuric acid and hydrogen peroxide for ten min, rinsed with plenty of Milli-Q water, and dried with a stream of nitrogen before use. The mass of deposited material on the sensor surface was calculated from frequency changes with the Sauerbrey equation. For calculating the thickness of the polypyrrole coating, a density of 1.48 g·cm^−3^ was assumed [9].

### 2.3. Synthesis and Characterization of Imprinted Polypyrrole Sensor

Polypyrrole was deposited onto the gold coated surface of piezoelectric quartz crystals or gold coated wafers through cyclic voltammetry of pyrrole monomer in the presence of clofibric acid in aqueous KNO_3_ solution or phosphate buffer solution (pH 7.0). The potential was cycled between −200 and +800 mV. After polymerization, the sensors were rinsed with ethanol and water and dried with a stream of nitrogen or were subject to washing under stirring (5 min–1 h) with 70% ethanol or PAD washing (15 min) in a mixture of ethanol and potassium chloride/hydrochloric acid (70:30, pH 2.5). NIPs were synthesized in the same way, but without clofibric acid.

The imprinted polypyrrole films were characterized with X-ray photoelectron spectroscopy (XPS) with a monochromatic Al Kα source. Binding energies refer to C1s (285 eV). Surfaces of MIPs and NIPs were analyzed with atomic force microscopy (AFM) (Nanosurf mobile S, non-contact mode) after polymerization, after washing and after binding tests. Measurements were made on 3–5 different areas on a sample (10 µm^2^, 1 line·s^−1^). Contact angle measurements were performed with the optical OCA 20 measurement system (Dataphysics) in the sessile drop mode. 15–20 drops of Milli-Q water per sample were analyzed (dosing volume 0.5 µL, 0.5 µL·s^−1^). For ellipsometry measurements (Horiba Jobin Yvon MM-16), polypyrrole samples made with 50, 120 and 240 cycles (40 mM pyrrole, 1 mM or no clofibric acid, phosphate buffer solution) were used. The thickness was fitted with the instruments DeltaPsi2 software. Zeta potentials of polymer films were measured with a SurPass electrokinetic analyzer (Anton Paar). In the cell, a 0.1 M solution of potassium chloride is passed with a pressure of max. 400 mbar between the surfaces of two samples with a gap of 100 µm. Samples were prepared on rectangular pieces of gold coated glass wafers (2 cm × 1 cm, 40 mM pyrrole, 50 cycles). Zeta potential measurements were performed after coating, after PAD washing with ethanolic potassium chloride solution containing hydrochloric acid with a pH of 3.0, and after PAD binding measurements with 30 µM clofibric acid in phosphate buffer solution with a pH of 7.0.

### 2.4. Binding Experiments

The sensor response to solutions with increasing clofibric acid concentrations was determined with QCM measurements according to Ebarvia *et al.* [39]. First water was pumped through the cell until a steady frequency reading was reached. Then, KNO_3_ solution was introduced into the cell until the frequencies were stable. The flow was stopped and the obtained value marked as F_KNO_3__. Clofibric acid solution (containing KNO_3_) was then pumped through the cell for some min. After stopping the pump, the stable frequency value was recorded (F_Cf_). The frequency shift for each concentration was calculated as the sensor response according to Equation (1):

∆F = F_KNO_3__ − F_Cf_ (1)

Binding experiments were also performed with PAD [13,29] in buffer solution and clofibric acid solution with stopped flow. A sequence of five potential pulses with a two-step waveform was applied, 1 s at 0 V and 1 s at 0.6 V *vs.* reference electrode. The sum of the 5th anodic and cathodic peak current was calculated as ∆I_0_. This was repeated every minute for 10 min (∆I_t_) in total. The sensor response was calculated from the current change Equation (2).

R_S_ = ∆I_0_ − ∆I_t_ (2)

**Figure A1:**
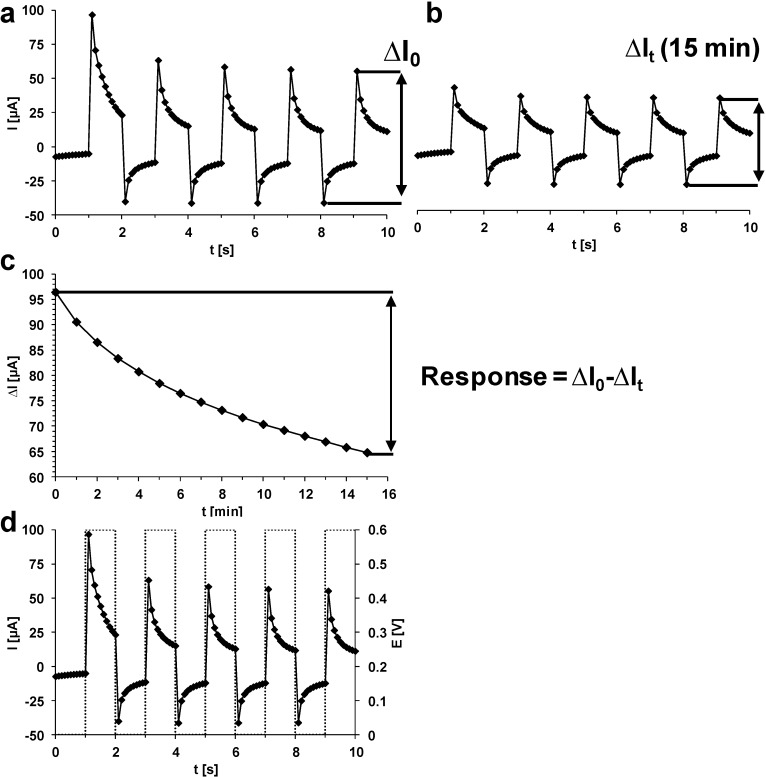
(**a**) PAD current response of polypyrrole during first application, last anodic and cathodic peak current gives ∆I_0_; (**b**) PAD current response during the 15th application, last anodic and cathodic peak current gives ∆I_15_; (**c**) Graph of ∆I_1–15_ against time, ∆I_0_ − ∆I_t_ gives sensor response. (**d**) Potential sequence and resulting current.

A graphic explanation of the entire procedure can be found in Appendix (Figure A1).

### 2.5. Washing Procedures

QCM sensors coated with polypyrrole were washed with ethanol or a mixture of ethanol and water (70% ethanol) in a PTFE holder with a magnetic stirrer. The solution was changed after 15 min. In another set of experiments, QCM sensors coated with polypyrrole were washed using a mixture of hydrochloric acid/potassium chloride and ethanol (1:1) (pH 3.5) under PAD conditions for 5, 10, 15, 30 or 60 min.

### 2.6. Selectivity Test

2,4-D, a common herbicide and structurally related to clofibric acid, and CBZ, an anticonvulsant used for treatment of epilepsy, were chosen for testing the selectivity of MIP. Like clofibric acid, CBZ is often not completely removed in waste water treatment plants and was found in environmental water bodies [40]. MIP and NIP coated QCM sensors were washed under PAD conditions for 15 min. Binding tests under PAD conditions were done with 30 µM solutions of 2,4-D and CBZ for 10 min and compared with the results of MIP and NIP binding tests with clofibric acid under the same conditions.

## 3. Results and Discussion

### 3.1. Influences of Template Concentration, Buffer, and Cycle Number on Polypyrrole Deposition

**Figure 2:**
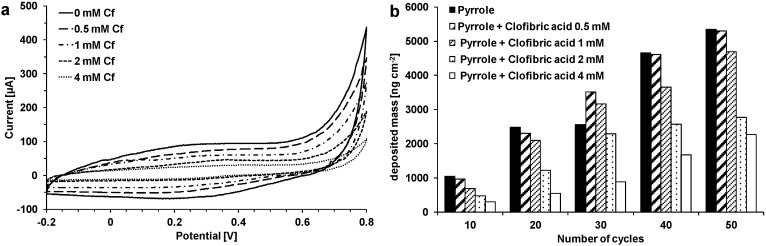
(**a)** Cyclovoltammograms during polymerization of pyrrole (10 mM); imprinting with clofibric acid (Cf) (0–4 mM) in KNO_3_ solution; (**b**) Influence of clofibric acid concentration on mass deposited after polymerization.

MIP and NIP sensor layers were polymerized electrochemically on gold coated quartz crystals via cyclic voltammetry (10–50 cycles). The deposition of the polymer was monitored by QCM-D, where the quartz crystal connected to a potentiostat is also the working electrode. During electropolymerization an oxidation peak appeared at 0.8 V. As no reduction peak occurred at the backward scan, the oxidation was irreversible. The first scan showed the highest oxidation peak. The increasing current in the range between 0.2 and 0.4 V indicated the buildup of polymer. It was observed that oxidation peaks were lower when clofibric acid was present in the polymerization solution. The influence of clofibric acid on the polymerization of pyrrole was further studied with different concentrations of clofibric acid between 0.5 and 4 mM and compared with NIPs (Figure 2a). With increasing fraction of clofibric acid present during polymerization the oxidation peak current decreased. Also, the current during the forward and the backward scan decreased with increasing concentration. This indicates inhibited polymerization and can also be observed by the QCM data. Figure 2b shows the polymer mass on the QCM sensors depending on the number of cycles and the clofibric acid concentration. With increasing cycle number, more and more polymer is deposited on the sensor. At the same time, an increasing concentration of clofibric acid leads to less mass deposition. For NIPs and MIPs with 0.5 mM of clofibric acid, between 1 µg·cm^−2^ (10 cycles) and 5.3 µg·cm^−2^ (50 cycles) of polymer were deposited on the sensor surface (thickness 7–36 nm). When 1 mM clofibric acid was used, 0.7 µg·cm^−2^ (10 cycles) to 4.7 µg·cm^−2^ (50 cycles) of polymer were deposited (thickness 5–32 nm). With 4 mM clofibric acid, the polymer mass decreased to 0.3 (10 cycles) to 2.3 µg·cm^−2^ (50 cycles) (thickness 2–15 nm). When the pyrrole concentration was increased to 20 mM, masses of 10–13 µg·cm^−2^ for NIPs (40 cycles) (69–87 nm) and masses of 9–11 µg·cm^−2^ (40 cycles) for MIPs (58–72 nm) were obtained. The highest deposited mass (18 µg·cm^−2^) was reached by using 0.5 mM clofibric acid, 20 mM pyrrole, and 80 cycles (122 nm).

**Figure 3:**
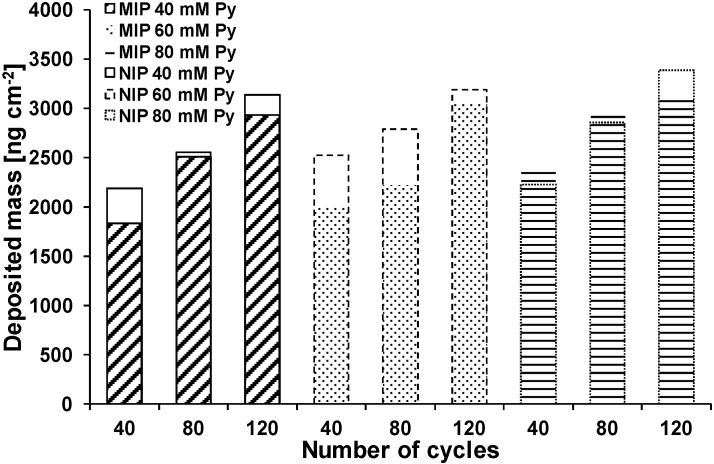
Deposited mass of polypyrrole obtained in phosphate buffer (blank columns NIP; filled columns MIP; in all experiments: 1 mM clofibric acid, Py: pyrrole).

As an aqueous solution of pyrrole and clofibric acid has a pH of 3.0, it was assumed that the reduced polymerization rate was caused by the formation of non-conjugated trimers of pyrrole, which inhibit the conductivity through incomplete conjugation (*cf*. Figure 1a) [41]. Another possible reason could be the precipitation of not dissociated clofibric acid (solubility 583 mg·L^−1^, *i.e.*, 2.7 mM, at 25 °C, estimated with EPI Suite v4.11) at the polymer surface, because due to clofibric acid’s pK_a_ of 2.8–3.2, at pH 3.0 only about 50% of the molecules are dissociated [42,43]. Anionic drugs with low solubility such as diclofenac (4.5 mg·L^−1^) and valproic acid (895 mg·L^−1^ at 25 °C) were also found to inhibit the polymerization of polypyrrole [44]. Therefore, the pH of the solution was increased to pH 7.0 by the use of phosphate buffer solution instead of KNO_3_. Pyrrole (40, 60, and 80 mM) was polymerized in buffer solution (pH 7.0) with or without 1 mM of clofibric acid on gold coated quartz crystals by cyclic voltammetry with 40, 80, and 120 cycles between −0.2 and 0.8 V. Because of the lower conductivity of phosphate buffer due to the lower mobility of phosphate compared to nitrate ions, the mass deposition in buffer solution was much lower than in KNO_3_ solution. In electrospray ionization–ion mobility spectrometry experiments, nitrate ions in methanol–water solutions had mobility values of 2.49 cm^2^·V^−1^·s^−1^, while for phosphate, hydrogen phosphate and dihydrogen phosphate values of 1.71, 1.91, and 2.16 cm^2^·V^−1^·s^−1^ were found [45]. With a 10 mM pyrrole solution in KNO_3_ solution, 4.7 µg·cm^−2^ of polypyrrole were deposited on the sensor surface, while with a 40 mM pyrrole solution in phosphate buffer only 2.2 µg·cm^−2^ of polypyrrole were formed at the same number of cycles (40). Furthermore, in the presence of clofibric acid, again mass deposition was decreased in most cases compared with NIPs (Figure 3). During oxidation of pyrrole, the release of protons (Figure 1a) could increase the acidity at the electrode interface [46]. This local acidity could lead to the precipitation of not dissociated clofibric acid and the formation of non-conjugated pyrrole trimers.

For nine MIPs made with 40 mM pyrrole and 120 cycles, 2.7 ± 0.2 µg·cm^−2^ (18 ± 1.3 nm) polymer were deposited on the sensor surface according to calculation with the Sauerbrey equation. For comparison, with ellipsometry measurements of samples made under the same conditions, thicknesses of 22 ± 1.5 nm were found for MIPs and 23 ± 1.9 nm for NIPs. This data indicates that the preparation of the thin-film MIP sensors can be reproduced effectively.

It is possible to overoxidize the polymer, which leads to the loss of conductivity through the introduction of oxygen containing groups such as carbonyl and carboxyl, and to dedoping [14]. On the other hand, overoxidation is viewed as a way to increase the quantity of functional groups, which can interact with the template, and to stabilize the cavities [47]. Overoxidation was not further studied in this case, because delamination of the film from the gold electrode occurred.

The following binding experiments were performed with samples polymerized in KNO_3_ (20 mM pyrrole, 40 or 80 cycles, 0.5 or 1 mM clofibric acid) or phosphate buffer solution (20 mM pyrrole, 40 or 80 cycles, 1 mM clofibric acid). For PAD binding experiments samples were prepared with pyrrole concentrations of 40, 60, and 80 mM and 40, 80, 120, and 240 cyclovoltammetric cycles.

### 3.2. Binding Experiments

The binding of analytes to molecularly imprinted polypyrrole had before been observed with QCM by several groups [11,15,16]. For an imprinted overoxidized polypyrrole film polymerized with constant current and a film thickness of about 80 nm, a mass change of about 1 µg·cm^−2^ was observed for the binding of L-aspartic acid [16] (uptake at constant potential of −0.4 V). For another imprinted overoxidized polypyrrole film polymerized with constant potential and a film thickness of about 120 nm a frequency decrease of ~220 Hz (1.2 µg·cm^−2^) was observed in the presence of 3 µM of dehydrocholate [15] (uptake at constant potential of +0.3 V). Overoxidized polypyrrole films polymerized with constant current and a film thickness of 300 nm for L-glutamic acid imprinted films and 650 nm for D-glutamic acid imprinted films showed mass increases of 9 µg·cm^−2^ (L-glutamic acid) and 2 µg·cm^−2^ (D-glutamic acid) in the presence of D- and L-glutamic acid, when they were polarized between +0.6 V and 0 V [14]. A caffeine imprinted polypyrrole sensor polymerized with constant current with a film thickness of ~2.5 µm showed a linear relationship between the frequency shift and the logarithm of caffeine concentrations between 0.5 mM and 50 mM (no potential) [11].

When the surface of a polypyrrole sensor polymerized in KNO_3_ came in contact with clofibric acid solution, a decrease of the QCM frequencies was observed. This could be explained by the uptake of clofibric acid by the polymer. In case of successful imprinting, the response of MIPs should be higher than the response of the corresponding NIP, but in this case the NIP (13 µg·cm^−2^) showed a higher response than the respective MIP (8.6 µg·cm^−2^). A nearly linearly response with a high frequency shift of up to 45 Hz (0.8 µg·cm^−2^) was obtained with the MIP with the highest mass (18 µg·cm^−2^), corresponding to a film thickness of 122 nm. Also, for polymers polymerized in phosphate buffer solution a decrease in frequencies was observed for MIPs. For NIPs an increase in frequencies was observed, but in one case a decrease of frequencies occurred. For MIPs, the sensor response is lower for polymers made of 20 mM pyrrole solution with a film thickness of 8–10 nm than for sensors made of 40 mM pyrrole solution with a thickness of 13–17 nm. Overall, the sensor response obtained with QCM must be considered invalid.

The concentrations of the clofibric acid solutions for binding might not be in the detection range of the sensor or the polymer might be too rigid to bind clofibric acid. A difference in the aforementioned studies is the film thickness, which is in most cases much lower in this study, but even for thicker films no clear difference between MIP and NIP could be obtained. Also, overoxidation for increasing the number of functional groups, which could interact with the template, was not used here due to delamination (*cf.* Section 3.1). Therefore, the method of pulsed amperometric detection was employed for polypyrrole made in phosphate buffer solutions and with clofibric acid as template. Applying a voltage to a conducting polymer adds or removes charges from the polymer backbone and induces the insertion or ejection of dopant ions, which changes the volume of the polymer [48]. During PAD the polymer is oxidized by a positive potential (0.6 V) and negative counter ions penetrate from the solution into the polymer, which begins to swell. Then, a lower potential (0 V) is applied, which leads to release of anions into the solution. The potential at 0 V is not sufficient to release all ions, which were incorporated during the pulse at 0.6 V, so the polymer might stay a bit more swollen than before. During the next pulses, the swelling of the polymer should increase and should allow the diffusion of more target molecules into the polymer [13]. The procedure and the method to extract a quantitative measure of sensor response (∆I) is described in Section 2.4 and illustrated in Appendix.

Three pyrrole (Py) concentrations and three different cycle numbers were chosen for polymerization of MIPs and NIPs. PAD measurements were performed on gold coated QCM sensors and polypyrrole sensors (40 mM Py) in buffer solution for 30 min. On gold, the current amplitude of the applied pulses was lower than 100 µA and did not change any more, when it reached 50 µA after 15 min. On polypyrrole, the current amplitude was at the beginning at 180 µA for a NIP (120 cycles) and at 260 µA for a MIP (240 cycles). A steady state at 100 µA (NIP) and 140 µA (MIP) is reached after 15 min. PAD measurements with clofibric acid solution on the gold surface of a QCM sensor showed low ∆I values around 25 µA compared to sensors coated with polypyrrole. The current generally decreases with every potential pulse over ten minutes and with increasing concentration of clofibric acid. This could be explained with the incorporation of clofibric acid into the polymer. For some sensors a linear decrease could be observed in the calibration graph (concentration *vs.* current change (∆I_0_ − ∆I_t_)), for example, MIP 40 mM Py 80 C and MIP 60 mM Py 80 C curves. However, also some NIPs showed this behavior (NIP 40 mM Py 80 C and NIP 60 mM Py 40 C).

Washing experiments were performed to find a procedure that can remove clofibric acid from the polymer, so that a sensor could be used repeatedly without loss of sensitivity. It was also tested if the application of PAD during washing could increase the sensor response. To prevent precipitation of salt in the narrow channels of the electrochemical flow cell, the fraction of ethanol was reduced to 50%. After the washing, binding tests were done with 30 µM clofibric acid. During the first PAD washing, the ∆I values decrease the most, while the decrease is lower during the second and third wash. A shorter pulse length of 0.5 s at 0.6 V gave a much lower sensor response (25 µA) to clofibric acid compared to 120 µA for a pulse length of 1 s. PAD washing was compared with wash under stirring. The highest response to 30 µM clofibric acid was obtained with PAD washing and a pulse length of 1 s. Also, this response occurred after the first washing, while for NIP, under stirring and with a shorter pulse length the highest sensor response occurred after the second wash.

**Figure 4:**
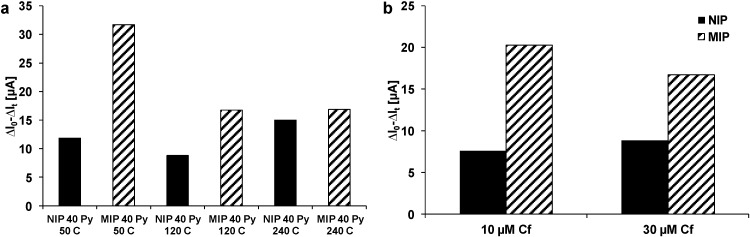
PAD sensor response of MIP and NIP to clofibric acid solutions. (**a**) PAD response to 30 µM clofibric acid solution of NIP and MIP prepared with 50, 120, and 240 cycles (40 mM pyrrole); (**b**) PAD response to 10 and 30 µM clofibric acid solution of NIP and MIP prepared with 120 cycles (40 mM pyrrole).

Therefore, for following experiments PAD wash was used. MIPs and NIPs were polymerized with 50, 120, and 240 cycles. Binding was tested with 30 µM clofibric acid (Figure 4a). The results show higher responses to the clofibric acid solution for MIPs than for NIPs, but the amount of non-specific binding is high. With increasing number of cycles, the response decreases for the MIPs. This could be explained by the thickness of the polypyrrole film, which increased from ~16 nm for 50 cycles to ~23 nm for 120 cycles and to ~29 nm for 240 cycles. A decreasing sensitivity and a decreasing specificity with increasing number of cycles was also observed with other polypyrrole coated electrodes and explained by slower diffusion of analyte molecules to the recognition sites [23,49]. Also with 10 µM clofibric acid, the difference between NIP and MIP was observable (Figure 4b). The response to PAD washing was for NIPs 153 ± 47 µA and for MIPs 170 ± 23 µA. Consequently, the feasibility of sensor fabrication via molecular imprinting electrochemical deposition of polypyrrole could be demonstrated, but the specificity (response for MIP *vs.* NIP) was strongly dependent on preparation and washing conditions and only modest success had been achieved. The possible reasons were investigated in more detail by film and surface analyses.

**Figure 5:**
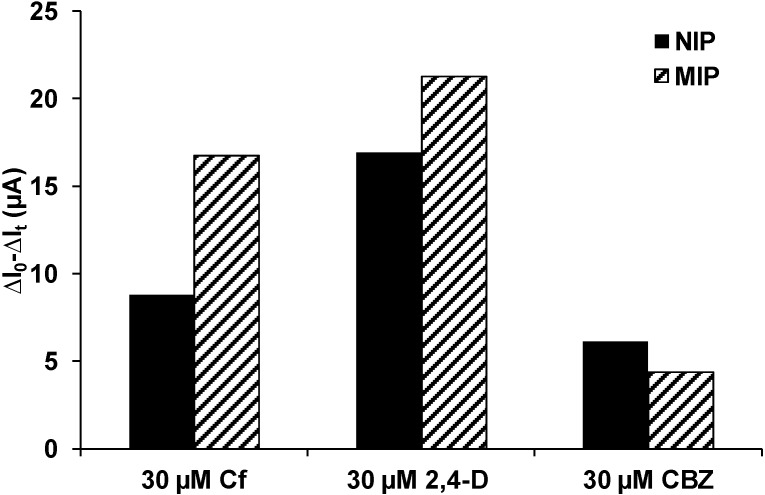
Selectivity test. PAD response of MIP and NIP prepared with 120 cycles (40 mM pyrrole) to 30 µM clofibric acid, 30 µM 2,4-D, and 30 µM carbamazepine.

Selectivity of the imprinted polymer and imprinting specificity were tested with 2,4-D and CBZ (Figure 5). The MIP showed a higher response to clofibric acid than the NIP (*cf*. Figure 4). The structurally closely related 2,4-D showed higher responses for both NIP and MIP. This higher sensitivity might result from the structure of 2,4-D, which consists of an acetic acid group instead of an isobutyric acid group. The hydrogen atoms in the acetic acid group might allow more hydrogen bonds between 2,4-D and polypyrrole than between clofibric acid and polypyrrole. The responses of MIP and NIP to CBZ were lower than to clofibric acid. The dibenzazepine structure of CBZ might form π–π-interactions with polypyrrole, but the carboxamide group might form less hydrogen bonds due to its resonance structures. However, when comparing the response to clofibric acid *vs.* that to CBZ, a significantly higher selectivity of the MIP (3.8) compared to the NIP (1.4) is obtained. The low response of the MIP to CBZ suggests imprinted cavities selective for clofibric acid and closely structurally related molecules; *i.e.*, this and the comparison with the NIP reveals some specificity of the imprinting process.

### 3.3. Surface Studies

To obtain information about the structure of the polymer, the template removal and the effects of rebinding, the polymer surfaces of NIPs and MIPs were analyzed. For XPS, samples were prepared on gold coated glass wafers or QCM sensors. After preparation samples were washed with ethanol (70%) and/or ultrapure water, acetonitrile or remained unwashed. Another set of samples was subject to PAD washing and binding experiments.

**Figure 6:**
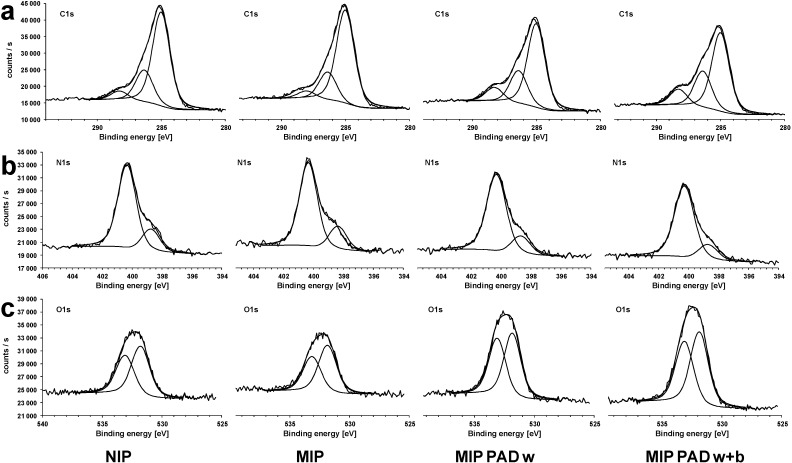
XPS spectra of NIP and MIP after polymerization, MIP after PAD washing, and MIP after PAD washing and subsequent clofibric acid binding of (**a**) C1s, (**b**) N1s, and (**c**) O1s.

The curve fitting of the C1s peaks for MIPs and NIPs had been done assuming three components at 285, 286, and 288 eV (Figure 6a). The peak at ~285 eV could be attributed to C-C bonds in the polymer chain, the peak at ~286 eV to C-N bonds and the peak at 288 eV could be explained by carbonyl groups [50]. The N1s peaks (Figure 6b) showed two components, the major peak at ~400 eV, which could be attributed to primary/secondary amine nitrogen (-N-H) [51,52], and a smaller peak at ~399 eV, which could be attributed to tertiary amine nitrogen (=N-), as the shoulder on the low energy side of the peak indicates an electron rich environment [53]. As polypyrrole is usually positively charged, if prepared in acid or neutral solutions, there should also appear a peak at ~402 eV, which is attributed to positively charged amine nitrogens [50,52,54]. The O1s signal (Figure 6c) consisted of two peaks at 532 eV and 533 eV, which could be attributed to C=O and C-O in the polymer backbone or to water or organic contamination [52].

**Figure 7:**
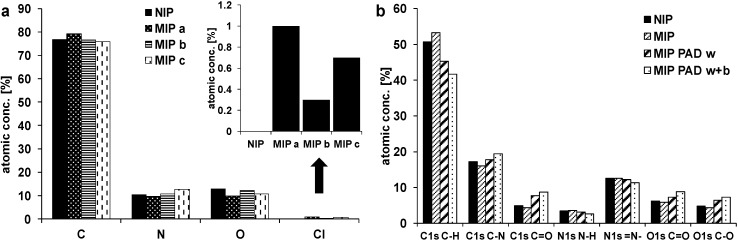
(**a**) XPS atomic concentration for NIP and MIP a (unwashed), MIP b (washed with ethanol), and MIP c (washed with acetonitrile); (**b**) XPS atomic concentration of C1s, N1s, and O1s components for NIP and MIP after polymerization, and for MIP after PAD washing and subsequent clofibric acid binding.

Chlorine was found in the MIP samples (Cl 2p ~200 eV), where the highest amount of chlorine was found for the unwashed sample (MIP a; Figure 7a). The lowest amount of chlorine was found for MIP b, which was washed with ethanol (70%) and water. Washing with acetonitrile (MIP c) was less effective, so that washing with ethanol (70%) was chosen for template removal in successive binding experiments. Samples, which were subject to PAD washing and PAD binding, contained a lower fraction of C-H groups and a higher fraction of C=O and C-O groups (Figure 7b). The XPS spectra show also higher oxygen peaks for these samples (Figure 6c). The repeated potential pulses during the PAD treatment might promote the oxidation of the polymer backbone or the swollen polymer might contain more oxygen from the solvents used during PAD. No chlorine was found in the NIP samples, which were polymerized without template molecules.

**Figure 8:**
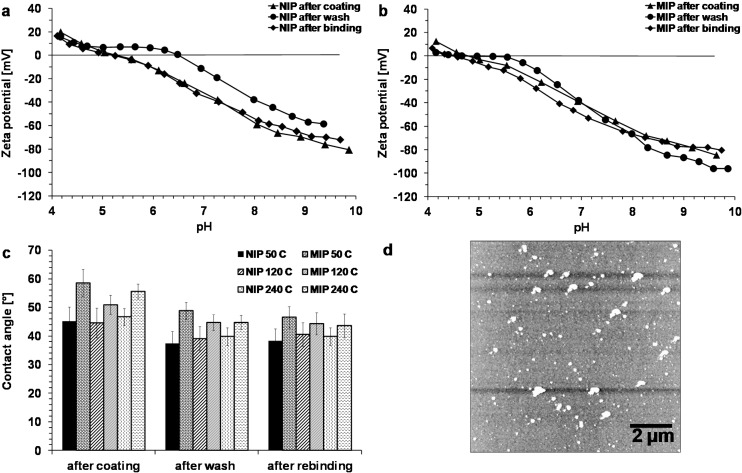
Surface analyses. (**a**, **b**) Zeta potential measurements of NIP (a) and MIP (b); (**c**) Contact angle measurements of MIP and NIP (40 mM pyrrole); (**d**) AFM 10 µm scan (mean fit) of MIP (40 mM pyrrole, 1 mM clofibric acid, 120 C).

Contact angle measurements were done with NIP and MIP of three different thicknesses after coating, after PAD wash and after binding (Figure 8c). For two gold samples, the contact angles were 82° ± 2° and 81° ± 3°. It was found that MIPs have higher contact angles (51° ± 3°–59° ± 5°) than NIPs (45° ± 5°–47° ± 3°). The incorporation of lipophilic clofibric acid (log P_OW_ 2.72) might make the polymer more hydrophobic. For both MIPs and NIPs, the contact angles decrease after washing, but the difference between MIPs and NIPs can still be observed (MIPs: 45° ± 3°–49° ± 3°, NIPs: 37° ± 4°–40° ± 3°). This is in accordance with the results from Apodaca *et al.* [55], who found higher contact angles for copolymers imprinted with bisphenol A than for non-imprinted copolymers. Polypyrrole films grown with various dopants had contact angles between 50° and 65° [56].

Zeta potential measurements (Figure 8a,b) show that the isoelectric point of the NIP after coating is at pH 5.3, while for the MIP the isoelectric point is at pH 4.8. The isoelectric point might be lower in the MIP due to the imprinting of clofibric acid, which itself is dissociated to 98%–99% at pH 4.8. After washing, the zeta potential of the NIP is positive for pH values between 4.2 and 6.5. This kind of plateau can also be observed with the MIP, where the zeta potential is positive for pH 4.2–4.7 and changes only slightly to −1 mV until pH 5.6. During washing, the amine group of pyrrole might be protonated by the hydrochloric acid in the washing solution. Also, the release of clofibric acid might reveal the positive charge of the polymer. After binding, the isoelectric point of the NIP changes back to the same value as after coating, and changes significantly to pH 4.5 for the MIP. The negative charge of clofibric acid can interact with the positive charge of the polymer (*cf*. Figure 1c). The low positive zeta potential values agree with the results of the XPS measurements, where no signal could be found for positively charged nitrogen atoms (*cf.* above). It could also be possible, that the electrostatic interaction with anions shifted the peak for positive charged nitrogen in the negative direction to the main peak of neutral nitrogen. After binding, the zeta potential curve of the NIP is nearly similar to the curve after coating, while the MIP shows more negative values. At pH 7.0, for the NIP the difference between the zeta potentials after coating and after binding is 3 mV (−32 mV *vs.* −35 mV), while for the MIP it is 10 mV (−40 mV *vs.* −50 mV). For polypyrrole particles with chlorine counter ions, positive zeta potentials in the range between pH 2–10 were found [57].

AFM images (Figure 8d) of MIP and NIP films polymerized in phosphate buffer solution revealed circular structures <1 µm on the surface. The roughness (rms) values were in the range between 6 and 8 nm and were slightly higher for MIPs than for NIPs. This is relatively smooth compared to other polypyrrole films synthesized with different dopants. A polypyrrole film doped with p-toluenesulfonic acid owned cauliflower-like structures with diameters of 10–20 µm [56]. A comparable roughness of 6 nm was found for polypyrrole films doped with poly(2-methoxyaniline-5-sulfonic acid) [58]. For imprinting, a rougher surface would be favorable, because more binding sites would be available on the surface, leading to higher binding capacity.

## 4. Conclusions

This study demonstrated the need for careful optimization of polymerization conditions for the imprinting of clofibric acid. It was found that polymerization is hindered by the presence of clofibric acid in KNO_3_ solutions and phosphate buffer solutions, presumably due to the release of hydrogen atoms during polymerization, which shift the pH near the electrode, so that undissociated clofibric acid might form. Several solvents were tested for removal of the template. With the use of 70% ethanol, the amount of chlorine found in XPS measurements decreased compared with water and acetonitrile. When binding was tested with PAD, it was concluded that the removal of the template after polymerization needed optimization, so washing was also tested under PAD conditions. Binding experiments showed then a higher response for the MIPs to clofibric acid than for the NIPs. Zeta potential and contact angle measurements also revealed differences between imprinted and non-imprinted polymers. Binding tests with 2,4-D and CBZ showed a pronounced selectivity of the imprinted polymer for clofibric acid *vs.* CBZ, but the response to 2,4-D was higher than the response for clofibric acid, presumably due to the structural similarities between the two molecules. Nevertheless, the non-specific binding was high. Other preparation conditions, such as pulsed potential or the roughening of the surface before polymerization [47] might allow the dedoping of the polymer with negative potential without delamination of the polymer. Furthermore, the use of other dopants, such as para-toluene s-e, which make rougher polypyrrole surfaces [56], should be carefully considered.
